# Neuronal LR11 Expression Does Not Differentiate between Clinically-Defined Alzheimer's Disease and Control Brains

**DOI:** 10.1371/journal.pone.0040527

**Published:** 2012-08-21

**Authors:** Kristen L. Sager, Joanne Wuu, Jeremy H. Herskowitz, Elliott J. Mufson, Allan I. Levey, James J. Lah

**Affiliations:** 1 Center for Neurodegenerative Disease, Emory University, Atlanta, Georgia, United States of America; 2 Department of Neurology, Miller School of Medicine, University of Miami, Miami, Florida, United States of America; 3 Department of Neurological Sciences, Rush University Medical Center, Chicago, Illinois, United States of America; 4 Department of Neurology, Emory University, Atlanta, Georgia, United States of America; Nathan Kline Institute and New York University School of Medicine, United States of America

## Abstract

Alzheimer's disease (AD) is the leading cause of dementia in the elderly. Because the pathological changes underlying this disease can begin decades prior to the onset of cognitive impairment, identifying the earliest events in the AD pathological cascade has critical implications for both the diagnosis and treatment of this disease. We previously reported that compared to autopsy confirmed healthy control brain, expression of LR11 (or SorLA) is markedly reduced in AD brain as well as in a subset of people with mild cognitive impairment (MCI), a prodromal clinical stage of AD. Recent studies of the LR11 gene *SORL1* have suggested that the association between SORL1 single nucleotide polymorphisms (SNPs) and AD risk may not be universal. Therefore, we sought to confirm our earlier findings in a population chosen solely based on clinical criteria, as in most genetic studies. Quantitative immunohistochemistry was used to measure LR11 expression in 43 cases from the Religious Orders Study that were chosen based on a final pre-mortem clinical diagnosis of MCI, mild/moderate AD or no cognitive impairment (NCI). LR11 expression was highly variable in all three diagnostic groups, with no significant group differences. Low LR11 cases were identified using the lowest tertile of LR11 expression observed across all cases as a threshold. Contrary to previous reports, low LR11 expression was found in only 29% of AD cases. A similar proportion of both the MCI and NCI cases also displayed low LR11 expression. AD-associated lesions were present in the majority of cases regardless of diagnostic group, although we found no association between LR11 levels and pathological variables. These findings suggest that the relationship between LR11 expression and the development of AD may be more complicated than originally believed.

## Introduction

Alzheimer's disease (AD) is the leading cause of dementia among the elderly, affecting one in eight individuals over the age of 65 and nearly half of all people over the age of 85 [1]. AD is a complex disease, with a wide range of genetic and environmental causes and a dense puzzle of underlying neuropathological changes. While the first clinical signs of disease typically emerge late in life, the pathological abnormalities that lead to AD often appear in the brain decades prior to the onset of cognitive impairment [2]. A burgeoning area of AD research has therefore focused on identifying genetic risk factors, early molecular changes, and behavioral endophenotypes in order to better identify those individuals at greatest risk for developing AD. Moreover, defining these early changes in the disease process can provide critical clues about potential therapeutic targets.

LR11, or SorLA as it is also known, is a multifunctional member of the lipoprotein receptor family that has recently emerged as a protein of interest in the neuropathology of AD. LR11 has been shown to play a crucial regulatory role in the processing of the amyloid precursor protein (APP) and may help to maintain low levels of the pathological Aβ peptide [3]–[6]. While LR11 protein levels in healthy brain are generally robust, LR11 protein expression is markedly reduced in the frontal cortex and hippocampus in AD [7]–[9]. In our previous study, we reported that LR11 protein expression is also reduced in the frontal cortex of a subset of individuals with mild cognitive impairment (MCI), similar to that seen in AD [10], suggesting that the loss of LR11 is an early step in the cascade of events underlying the development of this disease. Moreover, several studies report that single nucleotide polymorphisms (SNPs) in the LR11 gene (*SORL1*) are associated with an increased risk for developing AD [11]–[17]. Together, this makes LR11 a promising potential target for use as a diagnostic tool and as a site for therapeutic intervention.

An exciting but contentious area of research on LR11 is focused on understanding the relationship between polymorphisms in *SORL1* and the risk of developing AD. Although studies have shown a consistent loss of LR11 neuronal protein in AD brains, whether or not *SORL1* is a genetic risk factor for this disease remains controversial. While some studies have shown a significant association between SNPs in *SORL1* and an increase in AD risk, these relationships appear to be highly dependent on the clinical population being examined and are not universal [18]–[20]. For example, Liu *et al*. found no association between known *SORL1* SNPs and cognitive function in two separate Dutch cohorts [21]. Given these mixed results and the fact that most studies of LR11 protein expression in brain have been performed on samples from pathologically confirmed AD and healthy aged control cases, we sought to revisit our previous findings in brain tissue derived from a larger cohort that was chosen using selection criteria more similar to that used in most genetic risk factor studies; that is, with no restrictions in case selection based on pathology.

In this study, we quantified LR11 protein expression in 43 cases from the Religious Orders Study, a longitudinal investigation of aging and dementia [22], [23]. Cases for this study were chosen based on a clinical diagnosis of MCI, AD or no cognitive impairment (NCI) prior to death. Here, we report that in this larger population, low levels of LR11 protein expression were found in only a subset of the AD cases examined. Interestingly, a similar number of MCI and NCI cases were also found to have low LR11 expression, suggesting that a reduction in LR11 protein expression may not be a necessary precondition for the onset of cognitive impairment and the subsequent development of AD.

## Methods

### Case Material

The case demographics for the Religious Orders Study participants used in this study are reported in Table 1. The case materials for this study were obtained from the Religious Orders Study as part of a National Institute on Aging (NIA)-funded program project titled “Neurobiology of Mild Cognitive Impairment in the Elderly”. The use of Religious Orders Study materials for this project was approved by the Institutional Review Board of Rush University Medical Center. Each participant in the Religious Orders Study signs an informed consent form and an anatomical gift act at the time of enrollment. Participants also agree to annual neuropsychological and neurological evaluation as described previously [23]. Clinical diagnosis of AD follows criteria implemented by the Consortium to Establish a Registry for Alzheimer's Disease, with MCI and NCI classifications following the logic of these criteria [24]. Following death, each brain is sectioned into 1 cm slabs using a Plexiglas jig. Sections are then fixed in 4% paraformaldehyde and cryoprotected as previously reported [22]. Following fixation, diagnostic blocks are dissected from nine brain regions and cut into sections. AD pathological lesions (neuritic plaques, diffuse plaques and neurofibrillary tangles) are visualized by Bielschowsky silver stain. Hematoxylin and eosin stains are used to document chronic microscopic infarcts. The total numbers of each lesion present in a one square mm area viewed at 100× are counted in five brain regions (frontal, temporal, parietal and entorhinal cortices as well as the hippocampus). Using these counts, CERAD diagnoses [24]–[26], Braak stages of tangle pathology [27], and NIA/Reagan Consensus diagnoses [28], [29] are determined for each case. Our study consisted of 14 NCI, 15 MCI and 14 mild/moderate AD cases chosen from the Religious Orders Study cohort using the following criteria: age at death between 75 and 95 years, MMSE score of 10 or higher, post-mortem interval of 12 hours or less and a final cognitive evaluation less than 24 months prior to death. Only cases with a clinical diagnosis of NCI, MCI, or AD with no other cause of cognitive impairment were considered and cases with a clinical history of stroke and the presence of gross cerebral infarcts noted during autopsy were specifically excluded. Cases were matched on age, sex, education, and post-mortem interval to the extent possible. Although the three groups differed slightly in age (p = 0.029, Table 1), the difference did not reach the level of statistical significance set for this study (p<0.01, due to the large number of statistical analyses being run). Apolipoprotein E (apoE) genotyping was performed as described previously [30], [31], but apoE genotype was not considered in case selection.

**Table 1:** Clinical and demographic characteristics by diagnostic group.

Characteristics	NCI (n = 14)	MCI (n = 15)	AD (n = 14)	Total (n = 43)	Comparison by Group, *p*
Mean age at death ± SD (range), years	84.6±4.5 (78.1–92.8)	86.2±4.4 (79.4–93.6)	89.0±4.8 (76.4–94.5)	86.6±4.8 (76.4–94.5)	0.029^a^
Male sex, n (%)	5 (36%)	7 (47%)	4 (29%)	16 (37%)	0.67^b^
Mean education ± SD (range), years	17.6±4.0 (10–25)	17.8±3.6 (10–25)	18.2±3.4 (14–26)	17.9±3.6 (10–26)	0.99^a^
Mean postmortem interval ± SD (range), hours	5.4±2.4 (1.0–9.8)	6.2±2.6 (2.0–11.5)	4.9±2.0 (1.5–8.2)	5.5±2.4 (1.0–11.5)	0.49^a^
Subjects with *APOE* ε4, n (%)	1 (7%)	6 (40%)	6 (43%)	13 (30%)	0.072^b^
Mean MMSE ± SD (range)	28.1±1.5 (26–30)	27.1±2.6 (22–30)	18.8±5.8 (10–28)	24.7±5.6 (10–30)	p<0.0001^a^ (NCI, MCI>AD)
Mean GCS ± SD (range)	0.5±0.2 (0.2–0.9)	0.2±0.3 (−0.5–0.9)	−0.7±0.4 (−1.4–−0.2)	0.02±0.6 (−1.4–0.9)	p<0.0001^a^ (NCI, MCI>AD)

a Kruskal-Wallis test.

b Fisher's exact test.

### Immunohistochemistry

Free-floating, frozen cut 40 µm thick sections from the frontal cortex (corresponding to Brodmann's area 10) were labeled with polyclonal anti-sera to LR11 C-terminus generated against the peptide CEDAPMITGFSDDVPMVIA (Covance Research Products, Inc., Denver, PA), as used in our previous study [10]. This antibody has previously been used to immunoprecipitate full-length LR11 from post-mortem human brain, further establishing its specificity for LR11 [32]. Two to five sections were stained and analyzed per case. Sections were blocked with 8% normal goat serum, 0.1% Triton X-100 (Sigma Labs, St. Louis, MO), and 10 µg/ml avidin in Tris-buffered saline; incubated for 45 hours with anti-LR11; incubated for 1 hour with biotinylated goat anti-rabbit antibody (Vector Laboratories, Burlingame, CA) followed by avidin-biotinylated horseradish peroxidase (ABC reagent; Vector Laboratories) for 1 hour; and developed in 3,3′-diaminobenzidine. Three sections of frontal cortex tissue from a common case were included in each staining run as controls. One section was stained with either anti-calnexin (SPA-860; Assay Designs, Ann Arbor, MI) or anti-EEA1 (as2900; Abcam, Cambridge, MA) and served as a positive control. A second section was stained using the same protocol with the omission of a primary antibody to detect any non-specific label of tissue by the other reagents. No immunoreactivity was detected in any of these control sections. The final section was stained with LR11 CT and served as an internal control across staining runs. LR11 label of the internal control sections was highly consistent across staining runs.

### LR11 Quantitative Immunohistochemistry

LR11 neuronal immunostaining was measured using a quantitative approach as previously described [10]. Briefly, for each case, a total of five sampling regions were randomly selected from distinct areas of each stained section in order to ensure equal sampling across all tissue stained per case. Twenty sequential layer V pyramidal neurons were then imaged from each predetermined sampling region for that case, for a total of approximately 100 cells imaged per case (range 99–108). An average of 1 to 2 and a maximum of 6 cells were captured per image (Figure S1). Images were captured using a 100× oil immersion lens on an Olympus BX51 microscope fitted with an Olympus DP70 digital camera (Olympus America, Inc., Center Valley, PA). The selection of cells for imaging and the quantification of LR11 staining were performed by a single researcher blinded to clinical stage using the Metamorph image analysis program. Each cell in the field was outlined and a threshold was set at the most intense staining of the surrounding neuropil. All pixels within the cell that were stained more intensely than the background threshold level were considered positively stained for LR11. Because increasing LR11 staining intensity is reflected in increasing surface area stained positive for LR11 (Figure S2), the percentage of stained surface area for each cell was used as the LR11 measurement for each case.

### Protein extraction from brain tissue and immunoblotting

Post-mortem human brain homogenates were prepared as previously described [33]. Briefly, frontal cortex from each case was weighed individually (∼1 g) and homogenized (Dounce homogenizer) in PBS plus protease inhibitor cocktail (PIC) (Roche Diagnostics, Mannheim, Germany), Halt phosphatase inhibitor cocktail (Pierce, Rockford, IL), and lysis buffer containing 0.5% NP-40, 0.5% deoxycholate, 150 mM sodium chloride and 50 mM Tris, pH 7.4. Homogenized tissue was subjected to a 1000× g spin to remove debris. Cleared lysate was used for immunoblotting. Immunoblotting was performed as previously described [34]. To load equivalent amounts of tissue lysate per sample, protein concentration was determined by bicinchoninic acid (BCA) method (Pierce, Rockford, IL). Briefly, samples were resolved by SDS-PAGE and transferred to Immobilon-P membranes (Millipore, Bedford, MA). Blots were blocked with TBS plus blocking buffer (USB Corporation, Cleveland, OH) at room temperature for 45 min and probed with primary antibodies in TBS plus 0.1% Tween-20 plus blocking buffer overnight at 4°C. The next day, blots were rinsed and incubated with secondary antibodies conjugated to fluorophores (Molecular Probes/Invitrogen) for one hour at room temperature. Images were captured using an Odyssey Image Station (LiCor, Lincoln, NE), and band intensities were quantified using Scion Image. Antibodies used: mouse monoclonal LR11 (BD Biosciences, USA), rabbit polyclonal calnexin (Assay Designs, Ann Arbor, MI).

### Statistical Analyses

Clinical, demographic and neuropathological characteristics were summarized and compared across clinical diagnostic groups by Kruskal-Wallis test or Fisher's exact tests. Agreement between semi-quantitative scoring of LR11 expression by three blinded raters was assessed by weighted kappa. Correlation between the average semi-quantitative score from the three raters for each case and the average quantitative LR11 measurement for each case was assessed by Spearman rank correlation. Due to the considerable cell-to-cell variability within each case (intraclass correlation coefficient = 0.53), repeated measures analyses were used in all subsequent analyses of quantitative LR11 measures. Specifically, to estimate the average level of LR11 expression in each clinical diagnostic group and to assess the association between LR11 expression and clinical/neuropathological factors, we employed mixed models regression analysis with: random intercept, fixed effect covariate, Kenward-Roger denominator degrees of freedom, unequal variance assumption and unstructured covariance structure. For ease of interpretation, regression coefficients from these analyses, which quantify the direction as well as the magnitude of the effect (or association), are presented on the appropriate scale for the independent variable being examined. Statistical analyses were performed using SAS 9.2 (SAS Institute, Cary, NC) and Graphpad Prism 4.0 (Graphpad Software, San Diego, CA). To account for the large number of analyses performed in this study, the level of statistical significance was set at 0.01 (two-sided).

## Results

### LR11 Expression in the Frontal Cortex is Highly Variable in All Three Diagnostic Groups

**Figure 1:**
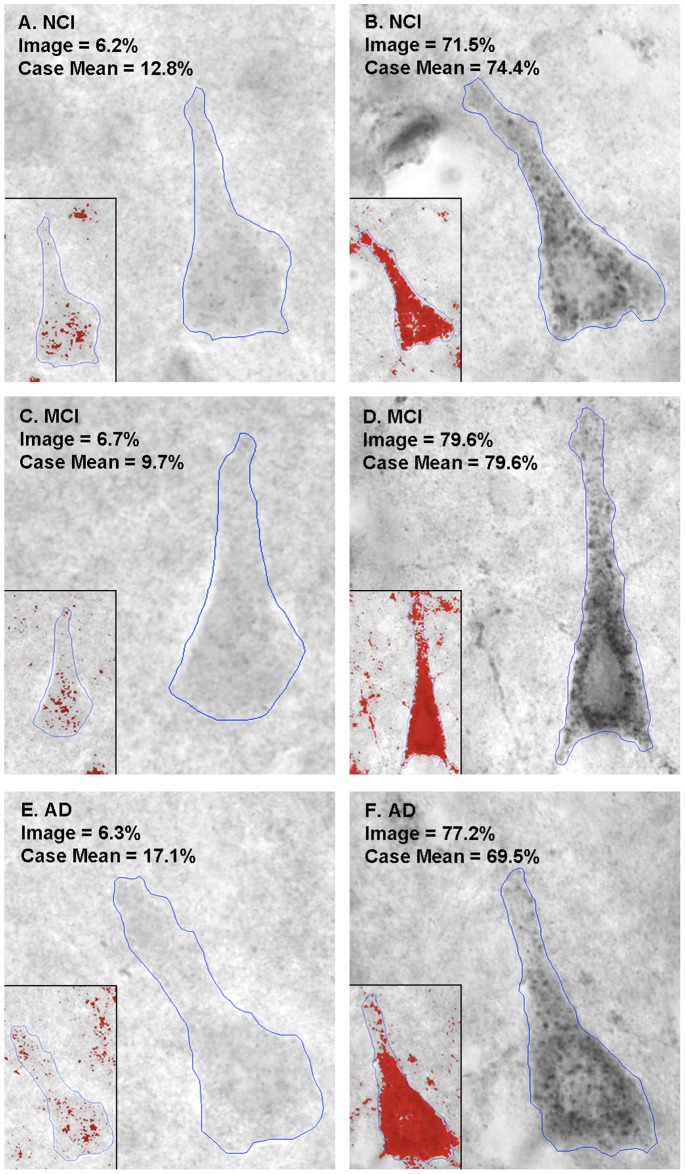
Representative images of LR11 immunostaining in each diagnostic group. LR11 expression in the frontal cortex was highly variable in all three diagnostic groups, as shown in these representative images demonstrating the range of staining in each diagnostic group. In all of the panels, the red overlay shown in the inset represents the pixels determined to be stained for LR11 for each pictured cell.

**Figure 2:**
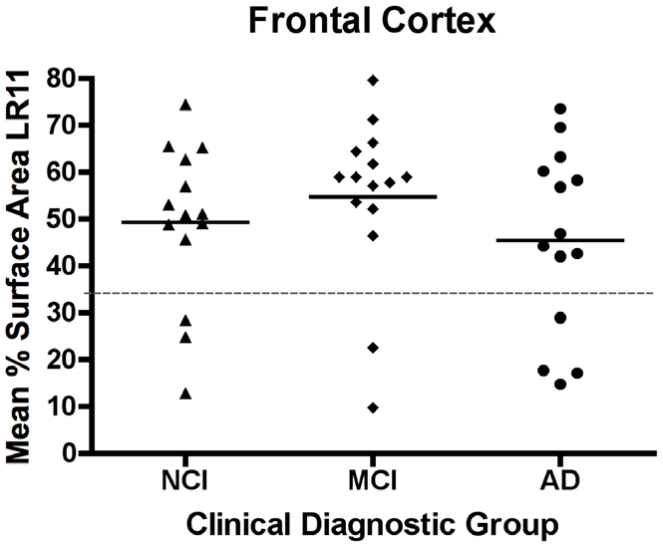
LR11 expression is highly variable in frontal cortex. Each point represents the mean LR11 expression for a single case and the short horizontal bars indicate the mean LR11 expression for each diagnostic group. There was no significant difference in mean LR11 expression between the NCI, MCI, and AD (p = 0.42). Moreover, a handful of cases in each group exhibited much lower LR11 expression than the majority of cases. Cases were classified as having low LR11 if the mean percent surface area stained for that case was in the lowest tertile of LR11 expression observed across all cases. This cut off (33%) is marked by the dotted line.

LR11 protein expression was measured in brain sections from the frontal cortex of 43 cases that were selected based on their final antemortem clinical diagnosis of NCI, MCI or AD. Neuronal LR11 immunostaining within each clinical group exhibited a wide range of expression levels, from robust punctate labeling of the cell body and proximal dendrites to a near absence of neuronal LR11 staining (Figure 1). LR11 expression in the NCI group ranged from 12.8% surface area to 74.4% surface area. LR11 expression in the other two groups was similarly varied, ranging from 9.72% to 79.6% in the MCI cases and 14.7% to 73.5% in the AD cases (Figure 2). There was no significant difference in the mean percent surface area across the three diagnostic groups (p = 0.42, Table 2). To better understand the distribution of LR11 expression profiles within each diagnostic group, we characterized all subjects with LR11 expression levels within the lowest tertile of LR11 expression observed across all cases as “low” LR11 cases. Using this cut off, we determined that 3 of 14 NCI cases, 2 of 15 MCI cases and 4 of 14 AD cases displayed low LR11 expression. There is no significant difference in the proportion of cases with low LR11 expression across diagnostic groups (p = 0.59, Table 2).

**Table 2:** LR11 Expression in frontal cortex is highly variable from case to case but comparable across clinical diagnostic groups.

	NCI (n = 14)	MCI (n = 15)	AD (n = 14)	Comparison by group, *p*
Mean % Surface Area LR11 ± SEM	49.2±4.6	54.6±4.6	45.4±5.2	0.42^a^
Number (%) of low LR11 cases	3 (21%)	2 (13%)	4 (29%)	0.59^b^

a By mixed models analysis.

b By Fisher's exact test.

**Figure 3:**
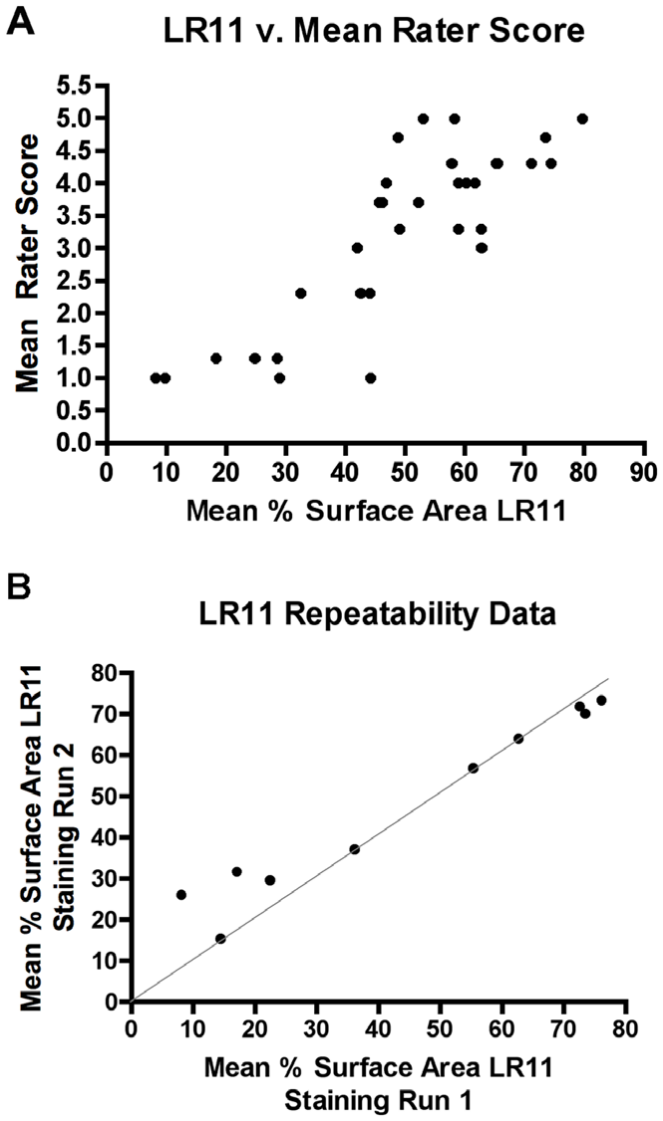
Verification of LR11 Quantitative Measures. (A) Three independent raters scored LR11 staining in 32 cases on a scale of 1 to 5 with 1 indicating little to no LR11 staining above background and 5 indicating strong LR11 staining. The quantitative LR11 measures and the average semi-quantitative scores from the three raters showed strong correlation (r = 0.73, p<0.0001), suggesting good agreement between the two methods. (B) To assess the consistency of the quantitative IHC approach, brain sections from 10 cases were re-stained and re-analyzed for LR11 expression in two independent experiments. Our method of measuring LR11 expression showed high reproducibility (intraclass correlation coefficient = 0.94). In the figure, proximity to the 45° line indicates closeness of the LR11 measures from the two runs.

These results were quite different from our previous findings showing a reduction in LR11 expression in AD [3], [7], [8] and MCI [10]. To confirm the validity of the current results, three independent raters blinded to clinical diagnosis and LR11 quantitative measurements scored LR11 immunostaining in the frontal cortex of the first 32 cases in the cohort on a semi-quantitative five point scale, with a score of 1 denoting no discernable cellular LR11 staining above background and a score of 5 denoting strong, consistent cellular LR11 labeling across the brain section. The three raters showed moderate agreement (average weighted kappa, κ = 0.56, range 0.37–0.69) and good correlation (Spearman r = 0.71–0.82, p<0.0001), similar to that seen in our previous study [10]. The average semi-quantitative score of the three raters also showed good correlation with the quantitative LR11 measures (Spearman r = 0.73, p<0.0001), confirming that our quantitative IHC approach produced a reliable assessment of qualitative LR11 staining intensity (Figure 3A).

To further confirm the reproducibility of these results, ten cases (including NCI, MCI and AD cases) were randomly selected from the current cohort and were re-stained and re-analyzed. All staining, imaging and analysis was done by the same researcher who was blinded to the previous results. The pairing between the values generated in the two independent staining runs was found to be highly reproducible (intraclass correlation coefficient = 0.94), further confirming the consistency of our methodology for measuring LR11 in this study (Figure 3B).

We also performed western blotting for LR11 using whole brain homogenates prepared from the frontal cortex, and similar to our findings in the imunohistochemical analysis, we found no significant or consistent differences between groups (Figure S3). It should be noted, however, that immunoblotting of brain homogenates does not discriminate between neuronal and non-neuronal sources of LR11 protein, and we have previously noted high expression of LR11 in glial cells and vascular structures in the same sections in which reduced neuronal LR11 expression is apparent [3], [7]. The persistence of non-neuronal LR11 expression is likely to confound detection of changes in neuronal LR11 expression by immunoblotting.

The relationship between frontal cortex LR11 expression and a series of clinical variables was examined to identify potential confounding factors. Cases were analyzed as one group, regardless of clinical diagnosis. A mixed models regression analyses revealed little to no association between LR11 expression and age, years of education or PMI. Regression coefficients quantifying these relationships are reported in Table 3. Likewise, there was no significant difference in LR11 expression between males and females or between apoE ε4 carriers and non-ε4 carriers.

**Table 3:** Frontal cortex LR11 is not associated with any clinical variables.

Variables	Unit of Change	Change in LR11 Expression^a^
Age	10 years	3.0±5.9% (*p* = 0.61)
Years of Education	5 years	1.5±3.9% (*p* = 0.71)
PMI	2 hours	1.1±2.4% (*p* = 0.65)
Gender	Male/Female*	−2.0±5.8% (*p* = 0.72)
*APOE* ε4 Genotype	Carrier/Non-Carrier*	−1.0±6.1% (*p* = 0.87)

a Data is presented as Mean ± SEM (*p*-value) of the regression coefficient estimated by mixed models analysis.

* Reference group for comparison.

### Relationship between Frontal Cortex LR11 Expression and Cognitive Impairment

In a previous study, we found that LR11 expression within the superior frontal cortex (Brodmann's area 9) was inversely correlated with the degree of cognitive impairment measured by a global cognitive score (GCS), which is a composite z-score calculated from 19 individual cognitive tests [10]. In contrast to our previous findings, we found no significant relationship between frontal cortex LR11 expression and MMSE score (p = 0.31) or GCS (p = 0.15) in the current cohort of cases. We also examined the relationship between LR11 and the z-scores for each of five separate cognitive domains to determine whether LR11 expression was related to impairment in a particular cognitive domain. Almost no change in LR11 expression was seen in response to a 0.5-point increase in z-score for episodic memory, semantic memory, perceptual speed, or visuospatial ability (Table 4). None of these relationships were found to be statistically significant (p values ranging from 0.33 to 0.99). A slightly larger increase in LR11 expression was seen in response to a 0.5-point increase in working memory z-score; however, this association was considered only weakly significant due to the large number of analyses performed on this dataset (p = 0.034). The difference in LR11 expression between amnestic and non-amnestic MCI cases also failed to reach statistical significance (p = 0.50, Table 4).

**Table 4:** Frontal cortex LR11 expression is not associated with cognitive performance or MCI subtype.

Variables	Change in LR11 Expression
MMSE Score	2.5±2.5% (*p* = 0.31)^a^
Global Cognitive Z-Score^b^	3.5±2.4% (*p* = 0.15)^a^
Episodic Memory Z-Score^b^	1.5±1.6% (*p* = 0.33)^a^
Semantic Memory Z-Score^b^	0.7±2.2% (*p* = 0.74)^a^
Working Memory Z-Score^b^	5.4±2.5% (*p* = 0.034)^a^
Perceptual Speed Z-Score^b^	−0.0±1.8% (*p* = 0.99)^a^
Visuospatial Ability Z-Score^b^	−0.4±2.0% (*p* = 0.83)^a^
MCI Subtype^c^	−6.9±9.9% (*p* = 0.50)

a Data is presented as Mean ± SEM (*p*-value) of the regression coefficient estimated by mixed models analysis for a 5 point (MMSE) or 0.5 point (z-scores) change in a given variable.

b Z-scores are computed based on the mean and standard deviation of a reference population. A 0.5-point difference in z-score indicates a difference of 0.5 standard deviation.

c Data is presented as Mean ± SEM (*p*-value) of the regression coefficient estimated by mixed models analysis for the comparison between amnestic (n = 5) and non-amnestic (n = 10) MCI cases, with the non-amnestic group serving as the reference group for comparison.

### Relationship between LR11 Expression and AD Pathology

It has long been observed that the pathological changes in the brain that result in AD first appear years and perhaps decades prior to the onset of cognitive impairment. Therefore, it is common to find both MCI and control cases that exhibit the pathological features of this disease, including amyloid plaques and neurofibrillary tangles (NFTs) [23], [35]–[38]. Accordingly, there was no difference in the frequency of AD pathological lesions between the three clinical diagnostic groups in this study in four of five brain regions examined (hippocampus, entorhinal cortex, inferior parietal cortex, and superior temporal cortex) (data not shown). Moreover, more than half of the NCI and MCI cases examined had at least moderate levels of neuritic plaques and diffuse plaques in the frontal cortex, just slightly less than the levels seen in the AD cases. Only the difference in NFT frequency in the frontal cortex between the three diagnostic groups reached statistical significance, with a significantly higher frequency of NFTs in the AD cases compared to NCI (p = 0.0084). It is worth noting that this difference is likely attributable to the complete absence of NFTs in all of the NCI cases in this cohort and that even in the AD cases, the frequency of NFTs was relatively low, with a mean frequency score of less than one on a 5-point scale (Table 5). Finally, only weakly significant differences between diagnostic groups were found in CERAD diagnosis (p = 0.018), Reagan diagnosis (p = 0.01) and Braak score (p = 0.020) (Table 5). Notably, the MCI group did not differ from either the NCI or the AD groups in any of these pathological measures. Together, these data suggest that many of our NCI and MCI cases may actually represent prodromal AD cases.

**Table 5:** All three diagnostic groups are pathologically AD-like.

Characteristics	NCI (n = 14)	MCI (n = 15)	AD (n = 14)	Total (n = 43)	Comparison by Group, *p* e
Neuritic Plaque Frequency^a^	2.1±1.4 (0–4)^d^	2.1±2.0 (0–5)	3.4±1.4 (1–5)	2.6±1.7 (0–5)	0.098
Diffuse Plaque Frequency^a^	2.8±2.2 (0–5)	2.4±2.2 (0–5)	4.6±0.9 (2–5)	3.2±2.1 (0–5)	0.012
NFT Frequency^a^	0 (0)	0.3±0.6 (0–2)	0.7±0.9 (0–3)	0.3±0.7 (0–3)	0.0084 (NCI<AD)
CERAD Diagnosis^b^	2.6±0.9 (1–4)	2.5±1.2 (1–4)	1.6±0.5 (1–2)	2.2±1.0 (1–4)	0.018
Reagan Diagnosis^c^	2.6±0.5 (2–3)	2.3±0.8 (1–3)	1.8±0.6 (1–3)	2.2±0.7 (1–3)	0.012
Braak Score	2.8±1.3 (1–5)	3.4±1.2 (1–5)	4.1±1.1 (1–5)	3.4±1.3 (1–5)	0.020

a Lesion frequency was reported on the following scale: 0 = none, 1 = sparse (1–2), 2 = sparse to moderate (3–5), 3 = moderate (6–12), 4 = moderate to frequent (13–19), 5 = frequent (20+).

b CERAD diagnosis was reported on the following scale: 1 = Definite AD, 2 = Possible AD, 3 = Probable AD, 4 = No AD.

c Reagan Diagnosis was reported on the following scale: 1 = High likelihood, 2 = Intermediate likelihood, 3 = Low likelihood, 4 = No AD.

d Data is presented as Mean ± SEM (range).

e Kruskal-Wallis test.

Given the AD-like levels of plaques and tangles and the similar variability in LR11 expression across all three diagnostic groups, we examined whether LR11 expression in the frontal cortex was related to the frequency of these lesions in this same brain region. No association was found between LR11 expression and the frequency of neuritic plaques (p = 0.64) or diffuse plaques (p = 0.45) in the frontal cortex. A weak association was observed between LR11 expression and the frequency of NFTs in the frontal cortex (p = 0.023), which is likely attributable to the large majority of cases in the cohort with high LR11 expression and no cortical NFTs. No significant relationship was seen between LR11 expression and CERAD diagnosis (p = 0.82), Reagan diagnosis (p = 0.79) or Braak score (p = 0.99). Regression coefficients quantifying these relationships are presented in Table 6.

**Table 6:** Frontal cortex LR11 expression is not associated with AD pathology.

Variables	Change in LR11 Expression^a^
Neuritic Plaque Frequency	−0.8±1.7% (*p* = 0.64)
Diffuse Plaque Frequency	−1.0±1.4% (*p* = 0.45)
NFT Frequency	−9.2±3.9% (*p* = 0.023)
CERAD Diagnosis	−0.6±2.7% (*p* = 0.82)
Reagan Diagnosis	1.1±4.0% (*p* = 0.79)
Braak Score	−0.02±2.2% (*p* = 0.99)

a Data is presented as Mean ± SEM (*p*-value) of the regression coefficient estimated by mixed models analysis for a one point change in the 5-point (lesion frequencies), 4-point (CERAD and Reagan Diagnoses) or 6-point (Braak Score) scale.

## Discussion

In 2004, we first reported that LR11 protein expression is reduced in the brains from patients with AD compared to aged control brain [7], a finding that was subsequently confirmed by several independent studies [3], [8]–[11]. In contrast to these earlier neuropathological studies, we did not observe significant differences in LR11 expression among AD, MCI, and NCI brains in the cohort examined in this study. Only about a third of the AD brains had low LR11 expression relative to the full set of cases. This is far less than what we observed in our previous study using Religious Orders Study cases in which LR11 levels were in the lowest tertile of observed LR11 expression in 100% of AD cases [10]. While these findings were unexpected in sporadic AD cases, we have previously observed persistent neuronal LR11 expression in familial AD brains [8], demonstrating that LR11 loss is not a universal element of AD pathology. Moreover, genetic studies have shown that certain SNPs in the *SORL1* gene may confer a modest increased risk for developing AD and that this relationship may be population-specific [18], [19], [20]. Despite several lines of evidence linking it to AD pathogenesis, evidence is emerging that reduced LR11 expression is not required for the development of AD.

Although a reduction in LR11 expression was not seen in the full cohort of AD cases examined, low LR11 expression in the frontal cortex was found in approximately 29% of the AD cases. Moreover, a similar proportion of cases in both the MCI and NCI diagnostic groups also showed reduced LR11 expression compared to the other cases in the cohort. Mechanistic *in vitro* studies have shown that LR11 plays an important role in promoting non-amyloidogenic processing of APP, thereby helping to maintain low levels of Aβ production. Moreover, reducing LR11 expression in cell culture and/or in animal models has been shown to result in increased Aβ levels [3], [5], [9], [32]. Given this important regulatory role, it stands to reason that a lack of LR11 protein expression in the human brain would be accompanied by enhanced or accelerated amyloidosis, leaving the individual at an increased susceptibility for progression to greater pathological stages of AD and possibly an increased risk for developing dementia. Additional work will be needed to test these hypotheses and to better clarify the temporal relationship between a change in LR11 expression and the onset of amyloid accumulation.

The current study was primarily designed to characterize LR11 expression in subjects with MCI. The MCI diagnosis was initially introduced as a clinical concept to separate cognitively impaired individuals from those with frank dementia [39]. Many, but not all, individuals diagnosed with MCI progress to greater stages of cognitive impairment, leading to an eventual diagnosis of AD. As a result, in research settings this diagnostic group has often been used to represent a state of prodromal AD. Brains from individuals with MCI have often been used in studies designed to identify “early” changes in AD. However, it is now widely acknowledged that cognitive impairment is a lagging indicator for the presence of disease, with the triggering events that lead to the development of AD potentially beginning decades before the first signs of cognitive difficulty [2]. Extensive cortical and hippocampal amyloid pathology and NFTs in the medial temporal lobe are common in post mortem MCI brains, making MCI and AD virtually indistinguishable upon autopsy [36], [40]. In fact, by the time cognitive changes are apparent, many of the neuropathological processes may have begun to plateau, including the production and deposition of Aβ [41].

In our previous study of LR11 using brains from the Rush Religious Orders Study [10], case selection was based on pathological criteria. This ensured that the NCI cases were free of amyloid pathology, making it a true disease-free control group. In the current study, brains from cognitively normal individuals were included in the NCI group irrespective of underlying AD pathology. As a result, both the NCI and MCI groups had significant AD pathology, suggesting that nearly all of the cases examined in the present cohort had already developed some disease related neurodegenerative pathology prior to death. Given this important difference in case selection as well as the relatively small sample sizes in these studies, additional work will be needed to clarify the relationship between LR11 expression, AD neuropathology, and disease progression, especially in the pre-clinical stages of the disease.

Notably, slight changes in tissue selection and staining protocols make it difficult to draw direct comparisons between our previous study and the work presented here. In particular, the tissue analyzed in our previous study was derived from the area of the frontal cortex corresponding to Brodmann's area 9 while the tissue used in this study was derived from Brodmann's area 10. While these two adjacent brain regions have very similar cytoarchitecture and are generally grouped together in functional analyses, it is possible that LR11 expression may be differentially expressed in these separate brain regions, both in the healthy brain and in AD brain. However, while an early report suggested that AD-associated LR11 loss occurs only in AD-vulnerable brain areas [3], a survey of additional brain regions (including the precuneus and primary visual cortex) indicates that this may not be true for neocortical regions. In the majority of cases examined, LR11 expression was either uniformly robust or uniformly reduced across all of cortical regions examined (unpublished observations). Therefore, it is unlikely that a slight change in brain region can account for the disparate findings between our two studies.

Finally, it is important to note that due to limitations in tissue availability, the size of this present study may be too small to detect statistically significant changes in LR11 expression between diagnostic groups, especially in light of our primary finding that only a third of the AD cases in this cohort have reduced LR11 expression. Given the growing mechanistic and genetic evidence linking LR11 to the development and/or progression of AD, a much larger study examining the relationship between LR11 expression and the appearance of AD pathological lesions, especially during the long preclinical stage of the disease, is certainly warranted.

## Supporting Information

Figure S1 **Illustration of sampling methodology.** (A) Following the mounting of the sections on a slide, five separate regions are pre-selected for imaging before viewing the slide under the microscope. At least one sampling region is chosen from all sections to ensure sampling from all stained tissue. (B) For each region selected, the section is first viewed at 10× magnification. A representative image is taken and an individual cell from the pyramidal cell layer of the gray matter is selected as the starting point for imaging, as noted by the yellow box. (C) Cells are imaged at 100×, with each image containing anywhere from one to eight cells. Twenty consecutive neurons within the pyramidal cell layer are imaged from each region for a total of 100 cells per brain region per case.
(TIF)

Figure S2 **Quantitative immunohistochemistry can detect a wide range of neuronal LR11 expression.** Panel A shows a cell with low LR11 expression, Panels B and C show cells with medium low and medium high LR11 expression, respectively, and Panel D shows a cell with very high LR11 expression. The red overlay shown in the inset of each image represents the pixels determined to be stained for LR11 for each cell. The number of pixels stained positively for LR11 is expressed as a percentage of the total number of pixels present within the outlined cell in the image.
(TIF)

Figure S3 **Total brain LR11 as measured by immunoblotting does not distinguish between diagnostic groups.** (A) Representative western blot showing total brain LR11 in four NCI cases, 3 MCI cases, and four AD cases. LR11 band intensities were quantified and the measurements were normalized to the calnexin loading control (shown) and a common internal control case that was included on each blot (not shown). (B) There was no significant difference in LR11 levels between the three diagnostic groups. (p = 0.19). It should be noted that immunoblotting of total brain LR11 is not an ideal means of identifying and quantifying differences in neuronal LR11 expression as robust levels of glial (C) and/or vascular (D) LR11 expression are frequently present even in the absence of neuronal LR11 expression, as shown in these representative images from two low neuronal LR11 cases. Arrows in panel C indicate glial cells stained for LR11. Arrows in panel D indicate blood vessels stained for LR11. In both panels C and D, arrowheads indicate neuronal cell bodies.
(TIF)
