# Supplementary figures and images for: Neuronal LR11 Expression Does Not Differentiate between Clinically-Defined Alzheimer's Disease and Control Brains
Source: PLoS One. 2012 Aug 21;7(8):e40527. doi: 10.1371/journal.pone.0040527 (PMC3424248; doi:10.1371/journal.pone.0040527)

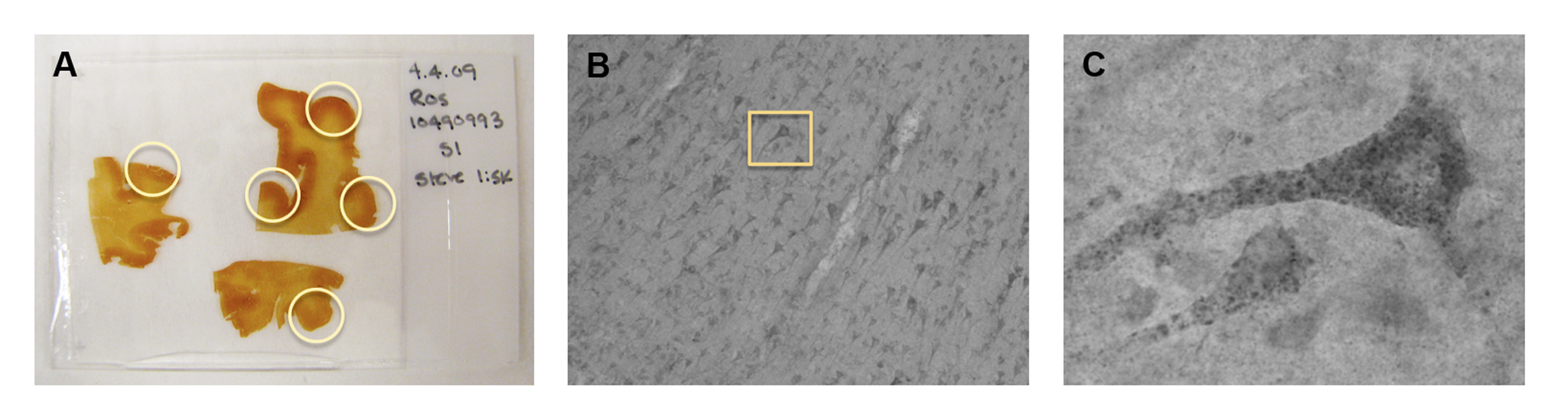

Supplement: Figure S1 — Illustration of sampling methodology. (A) Following the mounting of the sections on a slide, five separate regions are pre-selected for imaging before viewing the slide under the microscope. At least one sampling region is chosen from all sections to ensure sampling from all stained tissue. (B) For each region selected, the section is first viewed at 10× magnification. A representative image is taken and an individual cell from the pyramidal cell layer of the gray matter is selected as the starting point for imaging, as noted by the yellow box. (C) Cells are imaged at 100×, with each image containing anywhere from one to eight cells. Twenty consecutive neurons within the pyramidal cell layer are imaged from each region for a total of 100 cells per brain region per case. (TIF) [file pone.0040527.s001.tif]

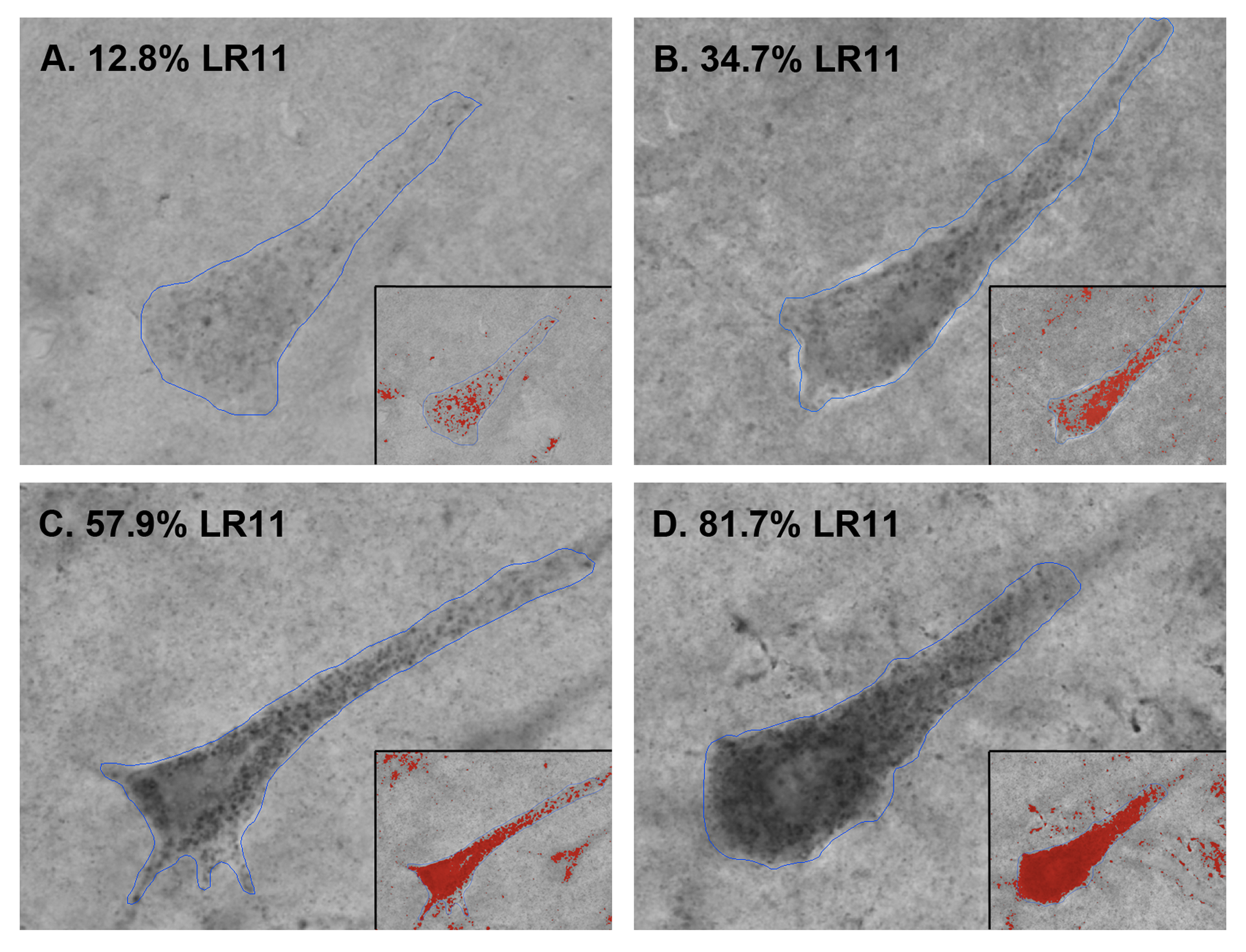

Supplement: Figure S2 — Quantitative immunohistochemistry can detect a wide range of neuronal LR11 expression. Panel A shows a cell with low LR11 expression, Panels B and C show cells with medium low and medium high LR11 expression, respectively, and Panel D shows a cell with very high LR11 expression. The red overlay shown in the inset of each image represents the pixels determined to be stained for LR11 for each cell. The number of pixels stained positively for LR11 is expressed as a percentage of the total number of pixels present within the outlined cell in the image. (TIF) [file pone.0040527.s002.tif]

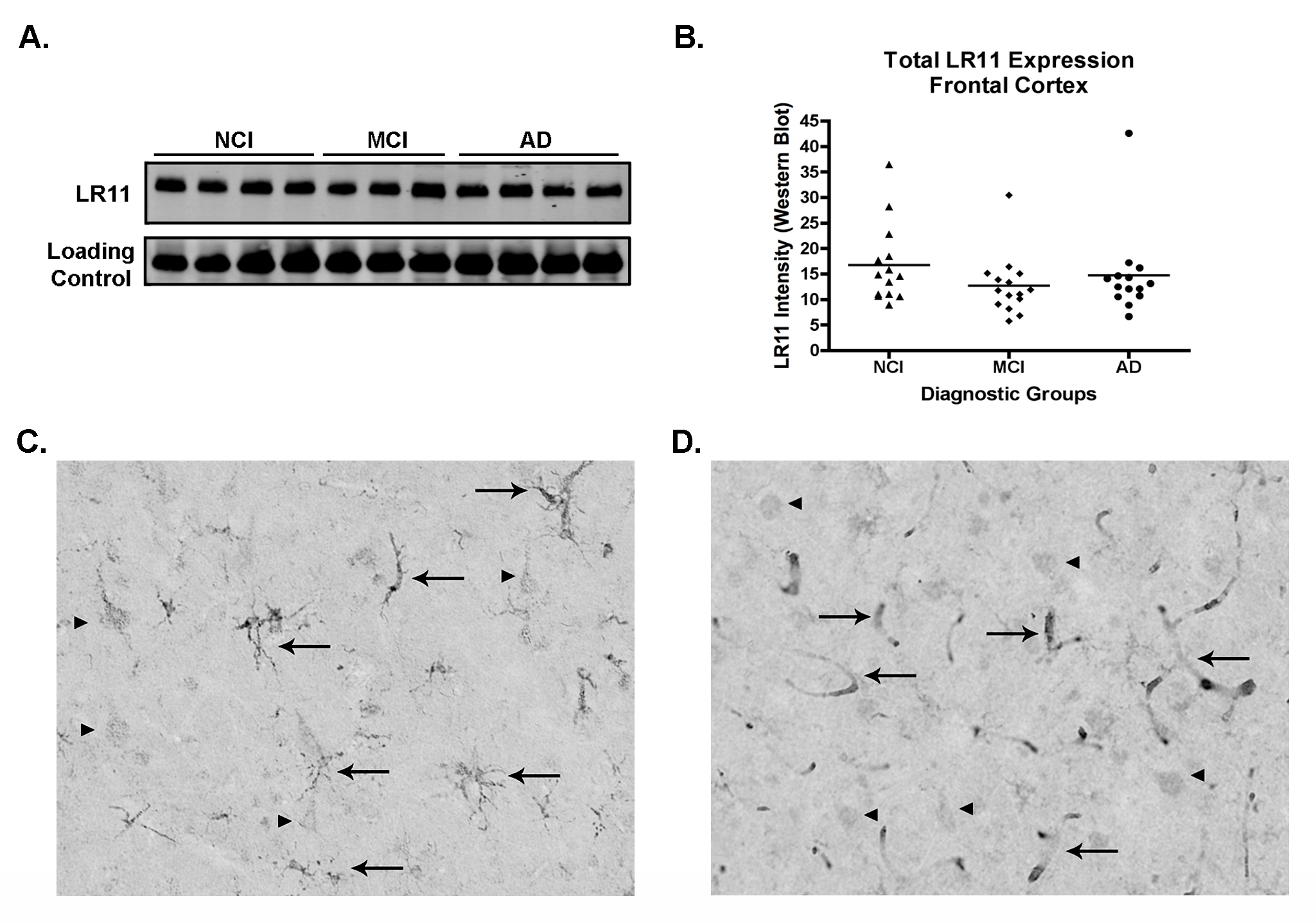

Supplement: Figure S3 — Total brain LR11 as measured by immunoblotting does not distinguish between diagnostic groups. (A) Representative western blot showing total brain LR11 in four NCI cases, 3 MCI cases, and four AD cases. LR11 band intensities were quantified and the measurements were normalized to the calnexin loading control (shown) and a common internal control case that was included on each blot (not shown). (B) There was no significant difference in LR11 levels between the three diagnostic groups. (p = 0.19). It should be noted that immunoblotting of total brain LR11 is not an ideal means of identifying and quantifying differences in neuronal LR11 expression as robust levels of glial (C) and/or vascular (D) LR11 expression are frequently present even in the absence of neuronal LR11 expression, as shown in these representative images from two low neuronal LR11 cases. Arrows in panel C indicate glial cells stained for LR11. Arrows in panel D indicate blood vessels stained for LR11. In both panels C and D, arrowheads indicate neuronal cell bodies. (TIF) [file pone.0040527.s003.tif]
